# Selective Angiography of Stimulant-Exposed Cardiac Donors Following Circulatory Death Does Not Impact Post-Transplant Outcomes

**DOI:** 10.3390/jcm14113809

**Published:** 2025-05-29

**Authors:** Clayton J. Rust, Ross Michael Reul, Helen Abadiotakis, Reshma Kodimerla, Joshua D. Preston, Supreet S. Randhawa, Michael E. Halkos, Muath M. Bishawi, Mani A. Daneshmand, Joshua L. Chan

**Affiliations:** Division of Cardiothoracic Surgery, Department of Surgery, Emory University School of Medicine, Atlanta, GA 30345, USA

**Keywords:** donation after circulatory death, cocaine, amphetamine, left heart catheterization, donor selection

## Abstract

**Background/Objectives**: Donation after circulatory death (DCD) has emerged to expand the heart-donor pool, but many DCD donors have risk factors such as cocaine or methamphetamine use. Stimulant use can cause coronary vasospasm and premature coronary artery disease, leading to routine donor coronary angiography (left heart catheterization, LHC) for coronary screening. However, performing LHC in DCD donors is challenging. We examined whether omitting LHC in stimulant-exposed DCD donors affects outcomes. **Methods**: A retrospective analysis was performed using the United Network for Organ Sharing (UNOS) database (2019–2024) to identify adult heart transplant recipients from DCD donors with documented cocaine or amphetamine use. Donors were stratified by whether antemortem LHC was performed. The primary outcome was 1-year recipient survival; secondary outcomes included graft failure and acute rejection. Kaplan–Meier survival curves and Cox regression analyses were performed. **Results**: A total of 485 DCD heart transplant recipients were identified; 135 (28%) donors underwent LHC and 350 (72%) did not. Recipient characteristics were similar between groups. No significant differences in 30-day (6% vs. 3%; *p* = 0.11), 90-day (6% vs. 3%; *p* = 0.21), or 1-year survival (7% vs. 6%; *p* = 0.48) were observed between the LHC and non-LHC cohorts. Graft failure and complication rates were also similar. However, among stimulant-exposed DCD donors with diabetes, an absence of LHC was associated with higher recipient mortality (HR 5.86, 95% CI: 1.57–21.87; *p* = 0.008). **Conclusions**: Routine donor coronary angiography may be unnecessary for stimulant-exposed DCD donors without additional risk factors. Omitting LHC did not compromise transplant outcomes. A selective LHC approach for high-risk DCD donors (e.g., diabetic donors) could safely expand the donor pool.

## 1. Introduction

Heart transplantation remains the gold-standard treatment for end-stage heart failure, yet the shortage of suitable donor organs is a persistent issue [1,2,3]. In recent years, the use of donation after circulatory death (DCD) heart donors has emerged as a viable strategy to expand the donor pool [4,5]. However, DCD hearts are often considered higher-risk grafts due to warm ischemic time and the lack of real-time functional assessment before procurement [6]. Technological advances in organ preservation, such as normothermic regional perfusion and ex vivo heart perfusion, have helped mitigate these concerns and enabled the increased utilization of DCD hearts [7,8]. The risk profile of DCD donors is further compounded by a high prevalence of stimulant use, particularly cocaine and methamphetamine, among potential donors [9]. Both cocaine and methamphetamine are known to have deleterious cardiovascular effects, including coronary vasospasm, accelerated coronary artery disease (CAD), and arrhythmias [10,11,12].

Given these risks, many transplant centers rely on pre-procurement coronary assessment with left heart catheterization (LHC) (i.e., coronary angiography) to screen for occult CAD in donor hearts. This approach is a common practice for expanded-criteria or high-risk donation after brain death (DBD) heart donors [13]. In DCD donors, however, the ethical and logistical challenges of invasive antemortem testing often preclude routine LHC. Obtaining explicit family consent for a procedure on a donor who is still legally alive and ensuring hospital resources (including interventional cardiology teams and catheterization labs) are available before circulatory arrest can pose substantial barriers. Furthermore, some clinicians and families may view invasive testing that does not directly benefit the donor as ethically problematic, making antemortem coronary angiography more difficult to justify in DCD settings. In Europe, ex vivo coronary angiography of donor hearts following procurement has gained popularity as an alternative, but this trend has not been widely adopted in the United States [14,15,16]. This disparity in practice raises an important question: Is routine LHC necessary in all stimulant-exposed DCD donors, or might this practice be unnecessarily limiting the donor pool?

This study aimed to assess whether the absence of antemortem LHC in stimulant-exposed DCD donors is associated with worse post-transplant outcomes. We hypothesized that, in the absence of additional cardiovascular risk factors, stimulant use alone may not necessitate invasive coronary imaging and that a more selective approach to donor angiography could expand the heart donor pool without compromising recipient outcomes.

## 2. Materials and Methods

### 2.1. Study Design, Data Source, and Study Population

This study utilized the Organ Procurement and Transplantation Network/United Network for Organ Sharing (OPTN/UNOS) national database. We performed a retrospective cohort study of adult heart transplant recipients from 2019 to 2024 who received hearts from DCD donors with a documented history of cocaine and/or methamphetamine use. Donors were stratified into two groups based on whether antemortem LHC (coronary angiography) was performed or not. Donors who underwent LHC but whose hearts were subsequently declined for transplant were not included due to dataset limitations.

### 2.2. Inclusion and Exclusion Criteria

All adult patients (<18 years of age) who underwent heart transplantation from 2019 to 2024 and had a history of cocaine and/or methamphetamine use were included. Pediatric patients, patients without data on drug use or the presence of left heart catheterization, and those without graft survival or mortality outcomes were excluded.

### 2.3. Data Collection and Variable Selection

Baseline donor and recipient characteristics were abstracted from the UNOS database, including demographics, comorbid conditions, and pre-transplant life support requirements such as extracorporeal membrane oxygenation (ECMO), intra-aortic balloon pump (IABP), mechanical ventilatory support, and renal replacement therapy. Donor variables included age, sex, body mass index (BMI), smoking history, heavy alcohol use, hypertension, diabetes, and a history of myocardial infarction.

### 2.4. Statistical Analyses

Categorical variables are reported as frequencies and percentages and were compared between LHC and non-LHC groups using the chi-square test. Continuous variables are summarized as median with interquartile range (IQR) or mean ± standard deviation, as appropriate, and were compared using Student’s *t*-test or the Wilcoxon rank-sum test based on data distribution. The primary outcomes of interest were recipient mortality at 30 days, 90 days, and 1 year post transplant. Secondary outcomes included graft failure at the same time points, the occurrence of acute rejection during the index hospitalization, incidence of post-transplant stroke, need for post-transplant dialysis, duration of post-transplant mechanical ventilation, and hospital length of stay.

Survival analyses were conducted using Kaplan–Meier methods for unadjusted comparisons of time-to-event outcomes. Cox proportional hazards models were constructed to assess associations between donor characteristics and recipient survival, stratified by donor LHC status. Variables with clinical relevance or with *p* < 0.10 in univariable analysis were included in multivariable Cox models (such variables included donor age, BMI, diabetes, hypertension, smoking history, and heavy alcohol use). Interaction terms were tested to evaluate effect modification by LHC status. Statistical significance was defined as *p* < 0.05. All analyses were performed using Python version 3.11 (Python Software Foundation, Wilmington, DE, USA) and Stata version 18 (StataCorp LLC., College Station, TX, USA).

## 3. Results

### 3.1. Donor and Recipient Demographics and Clinical Characteristics

A total of 485 heart transplant recipients who received stimulant-exposed DCD donor hearts met the inclusion criteria for analysis. Among these, 135 (28%) had donors who underwent antemortem coronary angiography (LHC group), and 350 (72%) had donors who did not undergo LHC (non-LHC group).

Recipient demographics, clinical characteristics, and waitlist times were comparable between the two groups, with no statistically significant differences observed in age, sex, race/ethnicity, socioeconomic status, comorbidities, or pre-transplant mechanical circulatory support requirements (Table 1). There were, however, significant differences in donor characteristics between groups (Table 2). Donors in the LHC group were older (38.8 vs. 31.4 years, *p* < 0.001) with a higher BMI (29 ± 7 vs. 28 ± 6 kg/m^2^, *p* = 0.05) compared to the non-LHC group. The prevalence of diabetes mellitus (8% vs. 3%, *p* = 0.02) and hypertension (21% vs. 12%, *p* = 0.02) were higher in the LHC group. Additionally, a greater portion of donors in the LHC group had a smoking history (37% vs. 17%, *p* < 0.001). Heavy alcohol use (47% vs. 37%, *p* = 0.06) and a history of myocardial infarction (3% vs. 1%, *p* = 0.08) were increased in the LHC group, but this did not reach statistical significance. Other donor factors, such as race, ethnicity, sex, and history of cocaine or amphetamine use, were similar between groups. Additionally, ischemic time did not differ between groups (4.97 vs. 5.03 h, *p* = 0.82).

### 3.2. Post-Transplant Outcomes

There were no significant differences in early post-transplant outcomes between the LHC and non-LHC groups (Table 3). Although post-operative dialysis was more common in the LHC group (20% vs. 15%, *p* = 0.08), the difference did not reach statistical significance. The incidence of acute rejection during the index hospitalization was also comparable (11.7% vs. 12.2%; *p* = 0.89) as were the rates of acute rejection requiring additional treatment within one year (10% vs. 16.5%; *p* = 0.37). Median length of stay (16 [13, 26] days vs. 16 [12, 23] days; *p* = 0.93) did not differ between groups.

**Figure 1:**
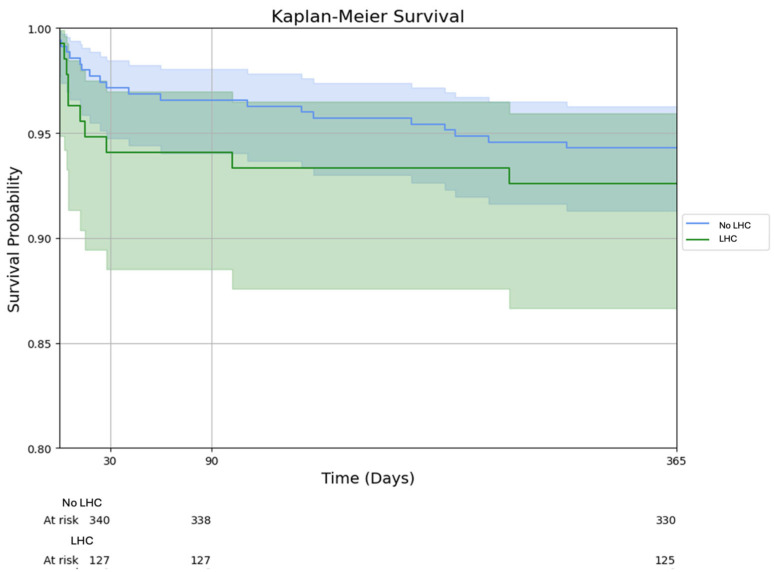
Kaplan–Meier survival curve stratified by donor left heart catheterization status. Survival curves are displayed for the LHC and non-LHC groups with corresponding 95% confidence intervals shaded. Time points at 30 days, 90 days, and 1 year post transplant are annotated. Risk tables show the number of patients at risk at each time interval for both groups.

Graft failure and survival outcomes are detailed in Table 4 and illustrated in Figure 1. Post-transplant survival was excellent in both cohorts at the 30-day (94.1% LHC vs. 97.15% non-LHC; *p* = 0.11), 90-day mortality (94.1% vs. 96.6%; *p* = 0.21), 1-year (92.6% vs. 94.3%; *p* = 0.48) timepoints without statistically significant differences. Graft failure (death or re-transplantation) within one year occurred in 7.4% of LHC and 6.0% of non-LHC cases (*p* = 0.57). There was a trend toward higher 30-day graft failure in the LHC group (6.7% vs. 3.1%; *p* = 0.079), but this difference was not statistically significant and diminished at later time points (90-day graft failure 6.7% vs. 3.7%, *p* = 0.16 and 1-year graft failure 7.4% vs. 6.0%; *p* = 0.57). These findings were also consistent when evaluating recipient mortality. When evaluating the impact of specific stimulant types on mortality, there was no significant difference identified on 1-year survival when stratified by donor drug category (93% cocaine-only donors vs. 92% methamphetamine-only donors vs. 94% combined stimulant use, *p* = 0.292).

### 3.3. Cox Regression Analysis

**Figure 2:**
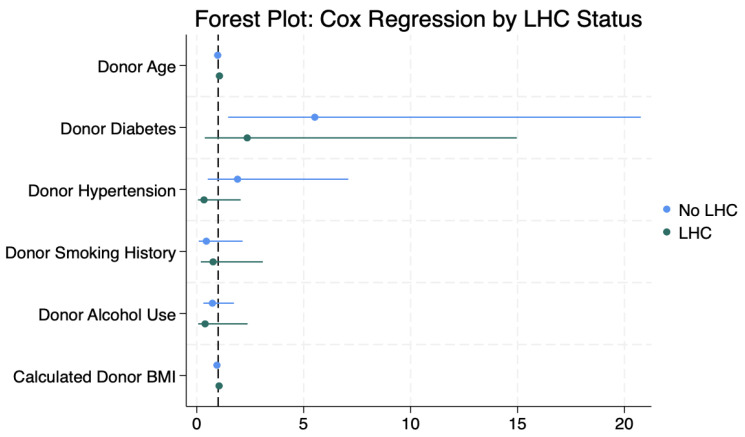
Forest plot of donor risk factors and recipient survival stratified by LHC status. Results are stratified by donor antemortem left heart catheterization (LHC) status. Points represent HRs, and horizontal bars indicate 95% confidence intervals. A vertical line at HR = 1.0 denotes no effect. Notably, donor diabetes in the non-LHC group is associated with significantly increased recipient mortality.

Cox regression stratified by LHC status (Figure 2) showed that donor diabetes was significantly associated with increased mortality in the non-LHC group (HR 5.86, 95% CI 1.57–21.87; *p* = 0.008), but not in the LHC group (HR 3.04, 95% CI 0.46–20.19; *p* = 0.25), suggesting a potential protective role of angiography in diabetic donors (Table 2). No other donor risk factors, including age, hypertension, smoking, or alcohol use, were independently associated with survival.

## 4. Discussion

The recent re-emergence of DCD heart transplantation has expanded the donor pool and decreased waitlist times for heart transplant candidates, with early outcomes demonstrated to be similar to those of traditional DBD heart donors [5,7]. As DCD heart donation becomes more common, transplant teams face evolving considerations regarding optimal donor selection and management. DCD donor hearts are still considered an expanded-criteria donor source, meaning careful donor evaluation and optimized procurement techniques remain critical to ensure excellent recipient outcomes. Many factors influence the acceptance of a particular donor heart for transplantation, such as donor age, cause of death, organ size, imaging and lab results, and availability of invasive studies [17]. However, the DCD donation process introduces unique logistical and ethical constraints that can limit the use of invasive donor evaluations like coronary angiography.

Current ISHLT consensus guidelines recommend coronary angiography for all potential heart donors over 45 years of age or with risk factors for premature CAD (including diabetes, significant tobacco use, or illicit drug use) [1]. These recommendations were developed primarily for DBD donors, for whom the decision to perform invasive coronary angiography is at the discretion of the organ procurement organization or the procurement team. Cocaine and methamphetamine are well-documented contributors to premature CAD, vasospasm, and arrhythmias [10,11,12]. Compounding these risks, donors who suffer fatal drug overdoses frequently have a history of heavy smoking, which further increases their cardiovascular risk profile [18]. Traditional barriers to performing donor angiography include the additional cost and resource utilization, variability in hospital capabilities (not all donor hospitals can support invasive angiography on short notice), and the need for available interventional cardiologists. In the DCD setting, the donor is legally alive during the evaluation period prior to organ recovery; thus, additional considerations (such as the need for family consent and the primary treating physician’s goals of care) come into play when deciding whether to pursue an invasive test such as LHC. These additional DCD-related constraints may therefore have an impact on overall donor acceptance and usage.

In this study, we analyzed national OPTN/UNOS data on the utilization of donor coronary angiography in an at-risk subgroup of DCD donors and the associated transplant outcomes. We found that only 28% of stimulant-exposed DCD heart donors underwent antemortem LHC prior to organ procurement, compared to approximately 48% reported for historical stimulant-exposed DBD heart donors. This reflects more a common use of angiography in the DBD donor population, potentially due to decreased barriers. While no national registry tracks donor hearts that are offered but ultimately not transplanted, the lower proportion of transplanted DCD grafts accompanied by LHC may signal that a substantial number of DCD hearts are being declined, and therefore never implanted, when coronary angiography cannot be performed.

Our analysis confirms that LHC in DCD donors is being applied in a manner that aligns with traditional coronary-disease risk. In the UNOS cohort, donors who underwent angiography were, on average, significantly older and demonstrated a higher prevalence of diabetes mellitus, systemic hypertension, and active or former tobacco use, which have all been considered risk factors for occult coronary atherosclerosis. Crucially, when these clinical variables are accounted for, stimulant use alone does not emerge as an independent predictor of early graft failure or recipient mortality, underscoring our central finding that isolated stimulant exposure should not automatically trigger invasive coronary imaging.

The preferential deployment of LHC to sicker donors also provides context for the nonsignificant (but numerically higher) 30-day mortality and graft-failure rates observed in the LHC cohort. This early hazard likely reflects the very comorbidity burden that prompted screening in the first place. By one year, survival curves for the LHC and non-LHC groups converge, further arguing that the initial divergence is attributable to baseline donor characteristics rather than to any intrinsic harm or benefit of angiography. An acknowledged major limitation is that national transplant registries do not capture donors whose organs were declined on the basis of LHC findings; consequently, we cannot quantify how many potentially salvageable hearts are excluded when angiography cannot be performed, nor can we assess the procedure’s true specificity for clinically significant CAD in DCD donors. Nevertheless, the available data collectively indicates that while LHC remains prudent for DCD donors with a substantial traditional risk-factor load, a blanket requirement for stimulant use alone is not supported and may unnecessarily restrict the donor pool.

Our findings support this selective strategy, as recipients of stimulant-exposed DCD hearts without additional risk factors had outcomes equivalent to those of donors who underwent LHC, whereas donors with significant risk factors (particularly diabetes) appeared to benefit from angiographic screening. These observations are supported by prior findings that elevated donor hemoglobin A1c correlates with worse post-transplant survival [19,20], and they align with current ISHLT recommendations for targeted angiographic screening in diabetic donors [1]. Based on these observations and existing guidelines, we propose a criterion for selectively performing donor LHC in stimulant-exposed DCD donors. Specifically, antemortem coronary angiography may be warranted for donors with traditional cardiovascular risk factors such as diabetes mellitus. Conversely, if a stimulant-exposed DCD donor has no additional risk factors besides drug use history, omitting LHC may be a reasonable, resource-conscious approach.

Several limitations of this study should be considered. First, the retrospective design and reliance on registry data introduce potential selection bias. We were unable to analyze data on DCD donor hearts that were evaluated but ultimately declined for transplantation, which could have provided insight into how angiographic findings (or a lack thereof) influenced organ acceptance decisions. It is likely that stimulant-exposed DCD donors who underwent LHC and still proceeded to transplantation had no significant coronary lesions; however, we do not know what donor factors or angiographic results led to organ declines in other cases. Second, the cohort of donors who did not undergo angiography had fewer traditional cardiovascular risk factors, which may have skewed the comparison of outcomes (i.e., a form of confounding by indication where lower-risk donors were selected to forgo angiography). The database does not capture preservation techniques (e.g., direct procurement vs. NRP), which prevents an analysis of their impact (although total ischemic time was reported and included in our study). Because the use of DCD heart transplantation is relatively new in the United States, one-year data currently represents the maximum interval with sufficient sample size. Future studies that extend follow-up and include larger, more robust subgroups, especially donors with diabetes or hypertension, will be essential to refine and clarify these observations. Finally, our analysis is constrained by the variables available in the UNOS database and may not capture all nuances of donor heart evaluation and post-transplant care (for example, granular data on coronary angiographic findings, or the details of rejection treatment protocols). Prospective studies or registry enhancements would be valuable to address these gaps.

## 5. Conclusions

Our data suggest that the absence of antemortem coronary angiography in stimulant-exposed DCD heart donors does not independently worsen recipient outcomes after transplantation. However, among DCD donors with additional coronary risk factors, particularly diabetes, the lack of invasive donor evaluation may be associated with inferior recipient survival. A more selective approach to donor coronary assessment in DCD heart transplantation by reserving LHC for those donors with traditional cardiovascular risk factors may allow for the optimal use of resources while maintaining excellent outcomes.

## Figures and Tables

**Table 1:** Recipient demographics and clinical characteristics stratified by donor left heart catheterization status. Baseline demographic, clinical, and life support characteristics of heart transplant recipients stratified by whether the donor underwent antemortem left heart catheterization (LHC).

Variable Stratified Variable	LHC (n = 135)	Non-LHC (n = 350)	Total (n = 485)	*p*-Value
Recipient				
Age (years)	54 ± 11	54 ± 12	54 ± 12	0.70
Sex				
* Male*	106 (79%)	294 (84%)	400 (83%)	0.20
* Female*	29 (21%)	56 (16%)	85 (17%)	-
Race/Ethnicity				
* White*	93 (69%)	227 (65%)	320 (66%)	0.47
* Black*	19 (14%)	70 (20%)	89 (18%)	-
* Hispanic*	20 (15%)	42 (12%)	62 (13%)	-
Insurance Type				
* Private*	70 (52%)	162 (46%)	204 (45%)	0.46
* Medicare/Medicaid*	62 (46%)	183 (52%)	245 (50%)	-
* Self-Pay*	-	-	-	-
Education Level				
* High school or less*	48 (36%)	133 (38%)	181 (37%)	0.29
* College or beyond*	77 (57%)	203 (58%)	280 (58%)	-
* * Employed				
* Yes*	22 (16%)	76 (22%)	98 (20%)	0.30
Body Mass Index (kg/m^2^)	28 ± 5	28 ± 5	28 ± 5	0.50
Diabetes	41 (30%)	113 (32%)	154 (32%)	0.64
Cerebrovascular Disease	6 (5%)	34 (10%)	40 (9%)	0.07
Dialysis	2 (1%)	7 (2%)	9 (2%)	0.77
Cancer	12 (9%)	33 (9%)	45 (9%)	0.89
Smoking History	56 (41%)	166 (47%)	222 (46%)	0.32
Life Support				
* ECMO*	3 (2%)	8 (2%)	11 (2%)	0.83
* IABP*	18 (13%)	54 (15%)	72 (15%)	0.57
* Mechanical Ventilator*	2 (1%)	4 (1%)	6 (1%)	0.83

ECMO = extracorporeal membrane oxygenation; IABP = intra-aortic balloon pump.

**Table 2:** Donor demographics and clinical characteristics stratified by left heart catheterization status. Baseline demographic and clinical characteristics of DCD heart donors with known stimulant use, stratified by whether the donor underwent antemortem left heart catheterization (LHC).

Variable Stratified Variable	LHC (n = 135)	Non-LHC (n = 350)	Total (n = 485)	*p*-Value
Donor				
Age (years)	39 ± 6	31 ± 7	33 ± 8	**<0.001**
Sex				
*Male*	112 (83%)	302 (86%)	414 (85%)	0.43
*Female*	23 (17%)	48 (14%)	71 (15%)	-
Race/Ethnicity				
*White*	107 (79%)	265 (76%)	372 (77%)	0.89
*Black*	8 (6%)	27 (8%)	35 (7%)	-
Hispanic	16 (12%)	49 (14%)	65 (13%)	-
Body Mass Index (kg/m^2^)	29 ± 7	28 ± 6	28 ± 6	**0.05**
Diabetes	11 (8%)	11 (3%)	22 (5%)	**0.02**
Hypertension	28 (21%)	43 (12%)	71 (14%)	**0.02**
Myocardial Infarction	4 (3%)	3 (1%)	7 (1%)	0.08
Smoking History	49 (37%)	57 (17%)	106 (22%)	**<0.001**
Heavy Alcohol Use	61 (47%)	124 (37%)	185 (40%)	0.06
Cocaine Use History	55 (41%)	200 (57%)	255 (52%)	**0.001**
Amphetamine Use History	54 (40%)	95 (27%)	149 (31%)	**0.001**

**Table 3:** Match run characteristics, intra-operative parameters, and post-operative outcomes stratified by donor left heart catheterization status.

Variable Stratified Variable	LHC (n = 135)	Non-LHC (n = 350)	Total (n = 485)	*p*-Value
Match Run				
Waitlist Time (Days)	22 [9, 167]	26 [8, 135]	25 [8, 147]	0.44
cPRA	21 ± 31	16 ± 24	18 ± 27	0.32
HLA Mismatch	5 [4, 5]	5 [4, 5]	5 [4, 5]	0.29
Sex Mismatch	20 (15%)	54 (15%)	74 (15%)	0.88
Ischemic Time (minutes)	309 [183, 381]	306 [217, 382]	307 [214, 382]	0.79
Outcomes				
Renal Replacement	27 (20%)	54 (15%)	81 (17%)	0.22
Cerebrovascular Accident	6 (4%)	13 (4%)	19 (4%)	0.71
ECMO	2 (1%)	11 (3%)	13 (3%)	0.31
Acute Cellular Rejection	14 (10%)	40 (11%)	54 (11%)	0.75
Treated for Rejection	3 (2%)	25 (7%)	28 (6%)	**0.04**
Length of Stay	16 [13, 27]	16 [12, 23]	16 [12, 24]	0.93

cPRA = calculated panel reactive antibody.

**Table 4:** Recipient and graft survival outcomes stratified by donor left heart catheterization status. Graft failure and mortality were assessed at 30 days, 90 days, and 1 year post transplant. Median graft and overall survival times are presented with interquartile ranges.

Variable Stratified Variable	LHC (n = 135)	Non-LHC (n = 350)	Total (n = 485)	*p*-Value
Graft Failure				
* * *30-day Graft Failure*	9 (7%)	11 (3%)	20 (4%)	0.08
* * *90-day Graft Failure*	9 (7%)	13 (4%)	22 (5%)	0.16
* 1-year Graft Failure*	10 (7%)	21 (6%)	31 (6%)	0.57
Recipient Mortality				
* * *30-day Mortality*	8 (6%)	10 (3%)	18 (4%)	0.11
* * *90-day Mortality*	8 (6%)	12 (3%)	20 (4%)	0.21
* * *1-year Mortality*	10 (7%)	20 (6%)	30 (6%)	0.48

## Data Availability

The original contributions presented in this study are included in the article/Supplementary Materials. Further inquiries can be directed to the corresponding author.

## Supplementary Materials

The following supporting information can be downloaded at: https://www.mdpi.com/article/10.3390/jcm14113809/s1, The complete raw data file and all computational analytical source code is available in Supplementary Material files.

## Abbreviations

The following abbreviations are used in this manuscript:

CAD	Coronary Artery Disease
CVA	Cerebrovascular Accident
DBD	Donation after Brain Death
DCD	Donation after Circulatory Death
ECMO	Extracorporeal Membrane Oxygenation
HR	Hazard Ratio
IABP	Intra-Aortic Balloon Pump
IQR	Interquartile Range
ISHLT	International Society for Heart and Lung Transplantation
LHC	Left Heart Catheterization
MI	Myocardial Infarction
OPTN	Organ Procurement and Transplantation Network
UNOS	United Network for Organ Sharing
