# Supplementary material for: Selective Angiography of Stimulant-Exposed Cardiac Donors Following Circulatory Death Does Not Impact Post-Transplant Outcomes
Source: J Clin Med. 2025 May 29;14(11):3809. doi: 10.3390/jcm14113809 (PMC12156159; doi:10.3390/jcm14113809)
Supplement: Supplementary file 1 [file jcm-14-03809-s001.zip › dcd_stim_do.pdf]

```
cd "/Users/chanlab/Desktop/STATA"

merge m:1 donor_id using DECEASED_DONOR_DATA
keep if _merge==3
save "/Users/chanlab/Desktop/STATA/merged_thoracic_donor.dta"


cd "/Users/chanlab/Desktop/STATA"
use merged_thoracic_donor.dta, clear
/// Select for first time isolated OHT ///
keep if num_prev_tx ==0
keep if organ =="HR"
tab organ, m

foreach var in txint txkid txliv txlng txpan txvca {
   tab `var', m
}
keep if multiorg !="Y"
keep if age >=18

/// Determine DCD Status ///
tab non_hrt_don, m
tab controlled_don, m
keep if non_hrt_don =="Y"
```

```
/// Determine Cocaine/Amphetamine Use ///

tab hist_cocaine_don, m

tab contin_cocaine_don, m

tab historydrugusecocaine, m

/// tab cocainedurationofuseunitid, m

tab cocainedurationofusevalue,m

/// tab cocainelastuseddate, m

tab cocaineuserouteid, m

tab historydruguseamphetamine, m

tab historyofdruguse,m

/// tab amphetaminesdurationuseunitid, m

tab amphetaminesdurationusevalue, m

/// tab amphetamineslastuseddate, m

tab amphetaminesuserouteid,m


g cocaine_meth =0

replace cocaine_meth =1 if hist_cocaine_don =="Y" | historydruguseamphetamine ==1 |historydrugusecocaine ==1 |contin_cocaine_don =="Y"

tab cocaine_meth, m


/// Determine Donor Angiogram ///

tab coronary_angio_don if cocaine_meth==1, m

tab coronary_angio if cocaine_meth==1, m

tab vessels_50sten if cocaine_meth==1


label define cath 1 "No" 2 "Yes, Normal" 3 "Yes, Abnormal" 4 "Yes, Stenosis <70%" 5 "Yes, Stenosis >70%"
```

```
label values coronary_angio cath


gen cath = 0 if coronary_angio == 1

replace cath = 1 if inlist(coronary_angio, 2, 3, 4, 5)

tab cath if cocaine_meth ==1, m


/// Cut cohort to only stimulant using donors ///

keep if cocaine_meth ==1

cd "/Users/chanlab/Desktop/STATA"

save "/Users/chanlab/Desktop/STATA/DCD_stimulant_use.dta", replace


/// Baseline Demographics & Comorbidities ///

label define insurance 1 "Private" 2 "Medicaid" 3 "Medicare FFS" 4 "Medicare" 6 "VA" 7 "Other Govt" 12 "Foreign Govt"

label values pri_payment_trr insurance

label define eth 1 "White" 2 "Black" 4 "Hispanic" 5 "Asian" 6 "Native American" 7 "Hawaiian" 9 "Multiracial" 998 "Unknown"

label values ethcat_don eth

label values ethcat eth

label define hxdm 1 "No" 2 "Yes, 0-5y" 3 "Yes, 6-10y" 4 "Yes, >10y" 5 "Yes, Unkknown" 998 "Unknown"

label values hist_diabetes_don hxdm


label define dm 1 "No" 2 "Type I" 3 "Type II" 4 "Other" 5 "Type Unknown" 998 "Unknown"

label values diab dm
```

/// Create binary variables for categorical variables ///

/// Recipient ///

```
g male_r =0
replace male_r =1 if gender =="M"
g white_r =0
replace white_r =1 if ethcat ==1
g black_r =0
replace black_r =1 if ethcat ==2
g hispanic_r =0
replace hispanic_r =1 if ethcat ==4
tab ethcat
tab white_r
tab black_r
tab hispanic_r


g insurance_r =.
replace insurance_r =1 if pri_payment_trr ==1
replace insurance_r =2 if pri_payment_trr ==2 |pri_payment_trr ==3 |
pri_payment_trr ==4 |pri_payment_trr ==6 |pri_payment_trr ==7 |pri_payment_trr ==12
tab insurance_r
tab pri_payment_trr
g private_r =0
replace private_r =1 if insurance_r ==1
g government_r =0
replace government_r =1 if insurance_r ==2
```

```
foreach var in cig_use cereb_vasc dial_after_list{
	gen `var'_num = .
	replace `var'_num = 1 if `var' == "Y"
	replace `var'_num = 0 if `var' == "N"
	replace `var'_num = . if `var' == "U"  // Keeps unknowns as missing
	label var `var'_num "`var' (1=Yes, 0=No, .=Unknown)"
	di "`var'"
	tab `var'
	tab `var'_num
}

gen hxdm_r = .
replace hxdm_r = 0 if diab == 1  // No
replace hxdm_r = 1 if inlist(diab, 2, 3, 4, 5)  // Any type of diabetes
replace hxdm_r = . if diab == 998  // Unknown
tab diab, m
tab hxdm_r, m

// Loop for 2x2 tabulation and Chi2 ///
	foreach var in male_r white_r black_r hispanic_r private_r government_r
cig_use_num ///
				cereb_vasc_num hxdm_r ecmo_trr iabp_trr
ventilator_trr ///
				dial_after_list_num {

	di "-----------------------------------"
	di "Tabulating `var' by cath"
```

```
tab `var' cath, column nokey chi2

}



// Loop for summary statistics and t-tests stratified by cath ///

foreach var in age bmi_calc days_stata1 days_stata2 days_stata3
days_stata4 days_stata5 ///

days_stata6 creat_trr end_cpra dayswait_chron
hlamis {


di "-----------------------------------"

di "Performing t-test for `var' between cath == 0 and cath == 1"

ttest `var', by(cath)

}


/// Donor ///

g male_d =0

replace male_d =1 if gender_don =="M"

g white_d =0

replace white_d =1 if ethcat_don ==1

g black_d =0

replace black_d =1 if ethcat_don ==2

g hispanic_d =0

replace hispanic_d =1 if ethcat_don ==4

tab gender_don

tab male_d

tab ethcat_don
```

```
tab white_d

tab black_d

tab hispanic_d


gen hxdm_d = .

replace hxdm_d = 0 if hist_diabetes_don == 1  // No

replace hxdm_d = 1 if inlist(hist_diabetes_don, 2, 3, 4, 5) // Any Yes

replace hxdm_d = . if hist_diabetes_don == 998  // Unknown

tab hist_diabetes_don

tab hxdm_d


foreach var in hist_mi hist_hypertens_don alcohol_heavy_don hist_cig_don
hist_cocaine_don contin_cocaine_don{

            gen `var'_num = .

            replace `var'_num = 1 if `var' == "Y"

            replace `var'_num = 0 if `var' == "N"

            replace `var'_num = . if `var' == "U"  // Keeps unknowns as missing

            di "`var'"

            tab `var'

            tab `var'_num

        }



    // Loop to tabulate categorical variables with Chi-square test

        foreach var in male_d white_d black_d hispanic_d hxdm_d hist_mi_num
hist_hypertens_don_num alcohol_heavy_don_num hist_cig_don_num
```

```
hist_cocaine_don_num contin_cocaine_don_num historydrugusecocaine
historydruguseamphetamine {

                        di "-----------------------------------"
                        di "Tabulating `var' by cath with Chi-square test"
                        tab `var' cath, column nokey chi2
                }

        // Loop to perform t-tests for continuous variables
                foreach var in age_don bmi_don_calc ischtime{

                        di "-----------------------------------"
                        di "t-test for `var' by Cath"
                        ttest `var', by(cath)
                }

/// Create recipient-donor gender mismatch variable ///
g genmis =.
replace genmis =1 if gender ==gender_don
replace genmis =0 if gender !=gender_don
tab gender gender_don
tab genmis
label define genmis 1 "Matched" 0 "Mismatched"
label values genmis genmis

/// Outcomes ///
```

```
gen mort30d = (ptime <= 30 & pstatus == 1)

gen mort1y = (ptime <= 365 & pstatus == 1)

tab mort30d cath

tab mort1y cath

g mort90d =0

replace mort90d =1 if pstatus==1 & ptime <=90

tab mort90d cath, col chi2



gen gfail30d = (gtime <=30 &gstatus ==1)

gen gfail1y = (gtime <=365 &gstatus ==1)

tab gfail30d cath

tab gfail1y cath

g gfail90d =0

replace gfail90d =1 if gstatus==1 & gtime <=90

tab gfail90d cath, col chi2


label define acr 1 "Yes, additional IS" 2 "Yes, no additional IS" 3 "No"

label values acute_rej_epi acr

tab acute_rej_epi


g acr =.

replace acr =1 if acute_rej_epi ==1 | acute_rej_epi ==2

replace acr =0 if acute_rej_epi ==3

tab acr

tab acute_rej_epi
```

```
foreach var in pst_dial pst_stroke trtrej1y{
                        gen `var'_num = .
                        replace `var'_num = 1 if `var' == "Y"
                        replace `var'_num = 0 if `var' == "N"
                        replace `var'_num = . if `var' == "U"  // Keeps unknowns as missing
                        di "`var'"
                        tab `var'
                        tab `var'_num
                }

foreach outcome in mort30d mort1y pstatus gfail30d gfail1y gstatus pst_dial_num
pst_stroke_num intubated_72hours ecmoposttx72 acr trtrej1y_num {
  di "-----------------------------------------------"
   di "Chi-square test for `outcome' by cath"
   tab `outcome' cath, column nokey chi2
}


di "-----------------------------------------------"
di "T-test for Length of Stay (LOS) by cath"
ttest los, by(cath)
```

```
di "----------------------------------------------"
di "Wilcoxon rank-sum test for LOS by cath"
ranksum los, by(cath)

stset ptime, failure(pstatus) // Setting up survival time for patient survival
sts test cath, logrank // Log-rank test for patient survival by cath

stset gtime, failure(gstatus) // Setting up survival time for graft survival
sts test cath, logrank // Log-rank test for graft survival by cath



/// Building multivariate regressions///
// Store significant variables (p < 0.2) for each outcome
// Define new binary predictors

// Define new binary predictors (ensure no space after the `///`)
local predictors "cath age male_r white_r black_r hispanic_r private_r government_r " ///
	"cig_use_num bmi_calc hxdm_r cereb_vasc_num creat_trr end_cpra " ///
	"dayswait_chron ecmo_trr iabp_trr ventilator_trr dial_after_list_num " ///
	"age_don male_d white_d black_d hispanic_d bmi_don_calc hxdm_d " ///
	"hist_mi_num hist_hypertens_don_num alcohol_heavy_don_num " ///
	"hist_cig_don_num hist_cocaine_don_num contin_cocaine_don_num"

di "`predictors'"
```

```
// Loop through each outcome
foreach outcome in mort30d mort1y gfail30d gfail1y pst_dial_num pst_stroke_num
intubated_72hours acr trtrej1y_num {
  di "-----------------------------------------------"
  di "Univariate logistic regressions for `outcome'"

  // Create an empty local macro to store significant variables
  local sig_vars

  // Check if outcome variable has valid observations
  capture count if !missing(`outcome')
  if r(N) == 0 {
    di "`outcome' has no observations, skipping..."
    continue
  }

  // Loop through each predictor
  foreach var of local predictors {
    capture confirm variable `var'  // Ensure variable exists
    if _rc == 0 {
      quietly logit `outcome' `var'

      // Get the p-value for the predictor variable
      quietly lincom `var'
      local pval = r(p)
```

```
      // If p < 0.2, add variable to the list
      if `pval' < 0.2 {
        local sig_vars `sig_vars' `var'
      }
    }
  }

  // Save the significant variables list for later use
  global model_vars_`outcome' "`sig_vars'"

  di "Significant variables for `outcome': `sig_vars'"
}

foreach outcome in mort30d mort1y gfail30d gfail1y pst_dial_num pst_stroke_num acr
trtrej1y_num {
  di "------------------------------------------------"
  di "Multivariate logistic regression for `outcome' using significant variables"

  // Ensure there are variables to include
  if "$model_vars_`outcome'" != "" {
    logit `outcome' $model_vars_`outcome', or
  }
  else {
    di "No significant variables for `outcome'. Skipping regression."
  }
}
```

```
/// KM Analysis ///
stset ptime, failure(pstatus)

sts graph, by(cath) ///
   title("Kaplan-Meier Survival by Cath Status") ///
   xlabel(0(30)370, angle(0)) ///
           tmax(365) ///
           plotopts(lcolor(emerald)) ///
   ylabel(0(.1)1) ///
   legend(order(2 "No LHC" 1 "LHC")) ///
   risktable (0 30 90 365, order (2 "No LHC" 1 "LHC"))


sts test cath


/// Cox Regression Analysis ///
stset ptime, failure(pstatus)
*------------------------------------------------------------
* Install coefplot if not already installed
*------------------------------------------------------------
ssc install coefplot, replace

*------------------------------------------------------------
```

```
* 1) Run the Cox regressions and store estimates for each cath group

*------------------------------------------------------------

stcox c.age_don hxdm_d hist_hypertens_don_num hist_cig_don_num alcohol_heavy_don_num bmi_don_calc if cath == 1, robust

estimates store reg_cath1


stcox c.age_don hxdm_d hist_hypertens_don_num hist_cig_don_num alcohol_heavy_don_num bmi_don_calc if cath == 0, robust

estimates store reg_cath0


*------------------------------------------------------------

* 2) Create a forest plot with custom variable labels and legend entries ("LHC" vs "non-LHC")

*------------------------------------------------------------

coefplot ///

   (reg_cath0, mcolor("90 147 239") ciopts(color("90 147 239")) label("No LHC")) ///

         (reg_cath1, mcolor(emerald) ciopts(color(emerald)) label("LHC")), ///

   eform drop(_cons) xline(1) ///

         rename (hxdm_d = "Donor Diabetes" ///

                        age_don = "Donor Age" ///

                        hist_hypertens_don_num = "Donor Hypertension" ///

                        hist_cig_don_num = "Donor Smoking History" ///

                        alcohol_heavy_don_num = "Donor Alcohol Use") ///

         title("Forest Plot: Cox Regression by LHC Status")
```
